# Effects of Breakfasts with Different Carbohydrate Contents and Glycemic Index Values on Postprandial Subjective Satiety and Plasma Gut Hormone Concentrations in Healthy Adults: An Exploratory Secondary Analysis of an Acute Randomized Crossover Controlled-Feeding Study

**DOI:** 10.3390/nu18213632

**Published:** 2026-10-31

**Authors:** Qian Ma, Wenjing Liu, Mengmeng Zheng, Wenxu Qin, Wenhui Yang, Huicui Meng

**Affiliations:** 1 School of Public Health (Shenzhen), Shenzhen Campus of Sun Yat-Sen University, Sun Yat-Sen University, Shenzhen 518107, China; maqian29@mail.sysu.edu.cn (Q.M.); liuwj87@mail2.sysu.edu.cn (W.L.); m15662683007@163.com (M.Z.); qinwx9@mail2.sysu.edu.cn (W.Q.); yangwh27@mail2.sysu.edu.cn (W.Y.); 2 Guangdong Province Engineering Laboratory for Nutrition Translation, Shenzhen 518107, China

**Keywords:** carbohydrate, glycemic index, gut hormone, satiety, visual analog scale, randomized crossover controlled-feeding trial

## Abstract

**Background/Objectives**: Data on the effects of low- versus high-glycemic index (GI) meals across various carbohydrate contents on postprandial subjective satiety and gut hormones are unclear. **Methods**: This study aimed to investigate the effects of low-carbohydrate (45%E) or high-carbohydrate (65%E) breakfasts with low-GI (≤55) versus high-GI (>70) values on postprandial subjective satiety and plasma gut hormone concentrations in healthy adults, and whether sex and the quantity and quality of habitual dietary carbohydrate intake influenced these effects. In this exploratory secondary analysis of an acute randomized crossover controlled-feeding trial, 15 healthy adults (19–25y) were randomly assigned to one of four breakfasts on separate days: high-carbohydrate low-GI, low-carbohydrate low-GI (LCLGI), high-carbohydrate high-GI, and low-carbohydrate high-GI meals. Postprandial subjective satiety scores were measured over 240 min following breakfasts, and area under the curve (AUC240) of fullness, hunger, desire to eat, and prospective eating scores were calculated. Plasma concentrations of ghrelin, glucagon-like peptide-1 (GLP-1), and glucose-dependent insulinotropic polypeptide (GIP) were measured at fasting and 240 min after breakfast. **Results**: Both low-GI breakfasts resulted in lower AUC240 (*P_GI_* = 0.0423) of fullness and higher AUC240 (*P_GI_* = 0.0291) compared to high-GI breakfasts. The LCLGI breakfast resulted in the highest AUC240 of hunger (*P_Interaction_* = 0.0178) and prospective eating scores (*P_Interaction_* = 0.0153) compared to the other three breakfasts. The postprandial plasma concentrations of ghrelin, GLP-1, and GIP were similar among the four breakfasts (all *p* > 0.05). **Conclusions**: These findings suggested a relatively unfavorable effect of the LCLGI breakfast on postprandial satiety, without influencing postprandial ghrelin, GLP-1, or GIP concentrations.

## 1. Introduction

Maintaining glucose homeostasis is essential for overall health status. Satiety, the postprandial suppression of appetite [1], is modulated by various gut hormones, such as ghrelin, glucagon-like peptide-1 (GLP-1), and glucose-dependent insulinotropic polypeptide (GIP), that subsequently influence glucose homeostasis [2,3]. These gut hormones are secreted in response to food and nutrient ingestion to coordinate appetite and metabolic signaling [3,4]. Elevated concentrations of circulating GLP-1 and GIP, together with suppressed ghrelin concentrations, synergistically inhibit appetite and maintain glucose homeostasis [3].

Dietary macronutrient composition is a critical determinant in satiety perception and postprandial gut hormone secretions. As the primary energy source in the human diet, both quantity and quality of dietary carbohydrate exert a pivotal influence on satiety and gut hormones [5,6]. Evidence regarding the impact of meal carbohydrate quantity on postprandial subjective satiety and gut hormones remains equivocal. Acute randomized controlled trials (RCTs) have reported higher postprandial subjective satiety within 3–7.5 h following meals with low-carbohydrate contents [31–35 percent of energy (%E)] compared to meals with moderate carbohydrate contents (54–60%E) [7,8,9], while other studies have reported null [10,11,12] or opposite findings [13]. Available data from acute RCTs have demonstrated higher postprandial plasma GLP-1 concentrations [11,12] and lower GIP [7,8,14,15] and ghrelin [9,10] concentrations within 2–8 h in response to meals with low-carbohydrate contents (30–38%E) compared to those with moderate or high-carbohydrate contents (54–84%E), while other studies have reported opposite findings [13,15,16].

Glycemic index (GI) was introduced to rank carbohydrate-containing foods based on their potential to raise postprandial blood glucose concentrations [17], and has been used as an indicator of carbohydrate quality. Although some studies have recognized the potential of low-GI breakfasts to enhance postprandial subjective satiety within 3–4 h following meals [18,19], other studies have demonstrated no significant difference in postprandial subjective satiety between low- and high-GI breakfasts when the energy contribution from dietary carbohydrates is either moderate (56–60%E) or low (~40%E) [20,21,22]. In terms of gut hormones, findings from acute RCTs have demonstrated that low-GI meals result in lower postprandial plasma GLP-1 concentrations [18] within 3 h following meals without influencing postprandial plasma ghrelin concentrations [23,24] within 2–6 h following meals in comparison to high-GI breakfasts when the energy contribution from dietary carbohydrates is either moderate (55–64%E) or low (~41%E). There is currently no study examining the effects of acute breakfast interventions with varying GI values on postprandial GIP concentrations.

Notably, there is a lack of systematic research directly comparing the effects of different combinations of carbohydrate content and GI. Only one study has investigated the effects of low-carbohydrate (~40%E) or high-carbohydrate (~60%E) meals with low-GI (GI value of 46–48) or high-GI (GI value of 72–76) values for an entire day on postprandial subjective satiety. The results found no significant differences in the mean area under the curve (AUC) of postprandial subjective satiety scores throughout the day among the four groups [25]. There is currently no study examining the effects of varying GI values on postprandial gut hormone concentrations.

The primary aim of this study was to assess the effects of low-carbohydrate or high-carbohydrate breakfasts with low-GI or high-GI values on postprandial subjective satiety and plasma concentrations of gut hormones, including ghrelin, GLP-1, and GIP in healthy adults. Exploratory analyses were conducted to examine whether these effects varied by sex and the quantity and quality of habitual dietary carbohydrate intake. We hypothesized that, with both carbohydrate contents, low-GI breakfasts would result in higher postprandial subjective satiety and lower ghrelin, higher GLP-1 and GIP concentrations than high-GI breakfasts.

## 2. Materials and Methods

### 2.1. Study Participants

Healthy adults aged 18–65 years with a body mass index (BMI) between 22 and 35 kg/m^2^ were enrolled in this randomized crossover study. The RCT was conducted at the Shenzhen Campus of Sun Yat-sen University. The participants were excluded based on the following criteria: fasting glucose concentration ≥ 6.1 mmol/L [26]; untreated hypertension; known chronic diseases (including cardiovascular diseases, diabetes, kidney, thyroid, gastrointestinal and liver diseases); use of medications known to affect glucose or lipid metabolism; use of nonsteroidal anti-inflammatory drugs or antihistamines; use of hormonal medications (e.g., anticoagulants, anabolic steroids, hydrocortisone); vegetarian; on a weight-loss diet; use of probiotics, multivitamins, fish oils, herbal supplements, or supplements containing dietary fiber; food allergies or other issues that could affect food intake; tobacco use; consume alcohol more than twice a day and refuse to stop during the study; weight gain or loss of more than 7 kg within six months before enrollment; unable to maintain body weight during the study; poor venous access; and unwilling to consume only the food and beverages provided during the study. The study was approved by the Sun Yat-sen University School of Public Health (Shenzhen) Biomedical Research Ethics Review Committee (SYSU-PH (SZ) ME [2024] 058), and written informed consent was obtained from all participants. The study was registered at the Chinese Clinical Trial Registry (ChiCTR2400093484; registered on 5 December 2024) and was conducted between 2024 and 2025.

### 2.2. Recruitment and Screening

Volunteers who responded to the recruitment poster underwent preliminary screening. Those who met the eligibility criteria were invited for an on-site visit. During this visit, participants received detailed information about the study design and underwent health screenings to confirm eligibility. Individuals who maintained their interest and fulfilled all eligibility requirements were subsequently enrolled in the study. A total of 38 volunteers were initially screened for eligibility, and 18 participants were enrolled in the study. Three volunteers did not complete all four interventions owing to personal reasons. A total of 15 participants completed four dietary interventions and were included in the final analysis (Figure 1A).

### 2.3. Study Design and Intervention

This study was a secondary analysis of an acute randomized crossover controlled-feeding trial. Each intervention protocol in the RCT consisted of a dinner followed by a breakfast, whereas the current study focused solely on the breakfast component. The dinner meal before the test day consisted of a standard moderate-carbohydrate meal with a medium-GI value (55.0%E from total carbohydrate, GI = 61.6). On the test days, participants were randomly assigned to receive one of four breakfast options based on a 2 × 2 factorial design on separate days. The test breakfasts included high-carbohydrate meal with low-GI value (HCLGI; 65%E from total carbohydrate, GI ≤ 55), low-carbohydrate meal with low-GI value (LCLGI; 45%E from total carbohydrate, GI ≤ 55), high-carbohydrate meal with high-GI value (HCHGI; 65%E from total carbohydrate, GI > 70), and low-carbohydrate meal with high-GI value (LCHGI; 45%E from total carbohydrate, GI > 70) [27]. Participants were provided with all foods and beverages. The foods included in the test breakfasts were summarized in Supplementary Table S1. The test breakfasts provided 645 kcal for men and 510 kcal for women, corresponding to 30% of the estimated total daily energy intake for Chinese adults [27]. Protein contributed approximately 20% of energy in all four breakfasts, whereas fat contributed around 35% and 15% in low- and high-carbohydrate breakfasts, respectively (Table 1). To minimize seasonal effects, all meals comprised only foods available year-round. The GI values of each breakfast were calculated as the weighted sum of the GI values of its component foods [28]. The GI values of individual foods were collected from the China Food Composition Tables [29] or food producers.

Each participant received all test breakfasts in a random order, separated by a washout period of 3–7 days. The randomization sequence was generated before the study, and allocation was determined by participants’ enrollment date and time. All research staff remained blinded to the testing order. Participants arrived at the trial center at 18:30 on the evening before the test day for a standard dinner. After an overnight fast, participants arrived at the trial center at 08:00 on the test day for fasting blood sampling and were instructed to complete a subjective satiety questionnaire on a subjective 100 mm visual analog scale (VAS), followed by a test breakfast that was required to be consumed within 10 min under supervision by trained staff. Starting from the first bite, participants completed a satiety questionnaire every hour to rate their hunger, fullness, desire to eat, and prospective eating. At 240 min post-breakfast, venous blood samples were drawn from the participants to measure postprandial subjective satiety-related gut hormone concentrations (Figure 1B). The 240 min postprandial time point was selected according to the original trial protocol to coincide with the final assessment of postprandial subjective satiety responses, as 240 min assessment periods have been commonly used in single-meal studies evaluating postprandial subjective satiety and gut hormone responses [30,31]. Participants were instructed to refrain from consuming any additional food or beverages except water after breakfast. Participants were asked to maintain their regular diet and exercise routine during the study, and they were instructed to abstain from alcohol and vigorous physical activity for 72 h before the test day. Sociodemographic characteristics were collected via questionnaires. Time and intensity of transportation, occupational, household, and leisure-time activities, as well as time of sitting, were assessed using the International Physical Activity Questionnaire (IPAQ), and calculated physical activity status was classified as “high”, “moderate”, and “low” based on established criteria [32]. Body weight, height, waist circumference, hip circumference, and blood pressure were measured.

### 2.4. Biochemical Analysis

Blood samples were collected in 5 mL ethylenediaminetetraacetic acid tubes, immediately placed at 4 °C for 30 min, centrifuged at 4 °C, 3000 rpm for 15 min to separate plasma, and then spun again at 4 °C, 10,000 g for 10 min. The plasma was then transferred to a clean polypropylene tube and frozen at −80 °C until analysis. Plasma concentrations of ghrelin, GLP-1, and GIP were measured using Bio-Plex Pro Human Diabetes Assays (#171A7001M, Bio-Rad Laboratories, Inc., Hercules, CA, USA) according to the manufacturer’s instructions. The intra-assay and inter-assay coefficient of variation (CV) specified by the manufacturer were 3–4% CV and 3–6% CV, respectively. GLP-1 concentrations were below the detection limit in some samples, and these values were imputed as half the limit of detection (1.84 pg/mL).

### 2.5. Habitual Dietary Intake Assessment

Habitual dietary intake over the past year was assessed using a semi-quantitative food frequency questionnaire (FFQ) administered by trained interviewers. The FFQ was developed in reference to the China Health and Nutrition Survey [33], including 10 food categories and 103 food items. For each food item, participants reported the frequency of habitual consumption (daily, weekly, monthly, or annually) over the past year and the amount consumed each time. The nutrient content of the edible portion of each food was calculated with the use of China Food Composition Tables [29,34].

Dietary GI and glycemic load (GL) values were calculated using previously established methods [35], and GI values for each carbohydrate-containing food were obtained from the China Food Composition Table [29] or International Tables of Glycemic Index and Glycemic Load Values [36]. Dietary carbohydrate to fiber ratio (CF) was calculated by dividing each participant’s mean daily total carbohydrate intake by their daily fiber intake. All nutrients and dietary GI, GL, and CF values were adjusted for total energy intake using the residual method [37]. Carbohydrate quality index (CQI) scores, including dietary GI, dietary fiber intake (g/d), solid carbohydrate to total carbohydrate ratio, and whole grain to total grain ratio, were calculated as described previously [38,39].

### 2.6. Subjective Satiety Assessment

Postprandial subjective satiety following the test breakfasts was assessed by the VAS satiety questionnaire prior to breakfast intake and every hour following breakfast intake for 4 h, consistent with the sampling interval used in previous studies [40,41]. Subjective feelings were rated on a 100 mm scale ranging from 1 (“not at all”) to 100 (“extremely”), with specific questions including [42]: (1) “How full do you feel right now?”; (2) “How hungry do you feel right now?”; (3) “How strong is your desire to eat right now?”; (4) “How much food do you think you can eat right now?”. Fullness was used to evaluate how full the participants were at the moment of investigation. Hunger was used to evaluate how hungry the participants were at the moment of investigation. Desire to eat was used to assess how much the participants wanted to crave food at the moment of investigation. Prospective eating was used to assess how much they thought they could eat at the moment of investigation. Participants chose to fill in the questions based on their actual feelings. Postprandial subjective satiety was evaluated by calculating the AUC from 0 to 240 min (AUC240) using the trapezoid method [43].

### 2.7. Statistical Analysis

Statistical analyses were performed using a per-protocol strategy, including only participants who completed all four interventions in the final analyses (*n* = 15). Data were assessed for normality using residual plots, and those that did not conform to a normal distribution were transformed using logarithmic or square root methods before statistical analysis. A repeated-measure two-way ANOVA model (PROC MIXED) was used to evaluate the main effects of carbohydrate, GI, and carbohydrate*GI interaction. The model included study period and sequence as covariates and participant as the random effect. Additionally, fasting subjective satiety scores were also included as a covariate for postprandial subjective satiety analyses of non-fasting time points and AUC240. For outcome measures exhibiting a significant carbohydrate*GI interaction, *post hoc* analyses were performed using the Tukey–Kramer method. Conversely, for outcome measures lacking a significant carbohydrate*GI interaction, *post hoc* analyses were conducted following a previously described method [44]. Hedges’ g was calculated using an established method to reflect effect size based on the current data [45]. Pearson or Spearman methods were used to analyze the correlation between postprandial subjective satiety and gut hormone concentrations, and between habitual dietary carbohydrate intake of the participants and postprandial subjective satiety as well as gut hormones concentrations according to the normal distribution of measurement data, and subgroup analyses of postprandial subjective satiety scores and gut hormone concentrations among the four groups were performed based on sex, %E from carbohydrate, CQI, and CF. Data were presented as least-squares means (LSmeans) with 95% confidence limits (CLs). Statistical significance was accepted at *p* < 0.05 (two-tailed). Statistical analysis was performed using SAS 9.4 (SAS Institute Inc., Cary, NC, USA) and R 4.5.0 (R Foundation for Statistical Computing, Vienna, Austria), and figures were depicted using GraphPad Prism 10.0 (GraphPad Software, La Jolla, CA, USA).

## 3. Results

### 3.1. Baseline Characteristics of Study Participants

Baseline anthropometric and lifestyle characteristics, dietary intakes, and fasting gut hormone concentrations were presented in Table 2. The mean age of participants was 22 ± 2 y, the mean BMI was 24.0 ± 1.7 kg/m^2^ (ranging from 22.1 to 27.7 kg/m^2^), and the mean waist circumference was 80.2 ± 4.2 cm (ranging from 73.3 to 86.1 cm). Seven participants (46.7%) were overweight. The mean total energy intake of participants was 2113.2 ± 139.6 kcal. The carbohydrate energy ratio of the participants was 50.3%. Additionally, the mean dietary GI and GL values, CF, and CQI score of the participants were 59.9, 146.1, 25.8, and 11.3, respectively.

### 3.2. The Effects of Low-Carbohydrate and High-Carbohydrate Breakfasts with Low-GI and High-GI Values on Postprandial Subjective Satiety Responses and Gut Hormone Concentrations

Both low-carbohydrate breakfasts had lower fasting fullness scores compared with both high-carbohydrate breakfasts (*P_Carbohydrate_* = 0.0490). Both low-GI breakfasts resulted in lower fullness scores than the high-GI breakfasts at 60 min (*P_GI_* = 0.0305) and 120 min (*P_GI_* = 0.0299) (Figure 2A). This resulted in lower AUC240 of fullness scores following low-GI breakfasts compared to high-GI breakfasts (*P_GI_* = 0.0423) (Figure 2B).

Both low-GI breakfasts had lower fasting hunger scores compared with high-GI breakfasts (*P_GI_* = 0.0410). The LCLGI breakfast resulted in the highest hunger scores compared to the other three breakfasts at 60 min (*P_GI_* = 0.0016, *P_Carbohydrate_* = 0.0100) and 120 min (*P_GI_* = 0.0029, *P_Carbohydrate_* = 0.0101). Both low-carbohydrate breakfasts resulted in higher hunger scores at 180 min compared with the high-carbohydrate breakfasts (*P_Carbohydrate_* = 0.0452) (Figure 2C). Collectively, the LCLGI breakfast resulted in the highest AUC240 of hunger scores in comparison to the other three breakfasts (*P_Interaction_* = 0.0178) (Figure 2D).

In terms of desire to eat scores, both low-GI breakfasts had lower fasting scores compared with high-GI breakfasts (*P_GI_* = 0.0109). Both low-GI breakfasts resulted in higher scores at 60 min (*P_GI_* = 0.0471) and 120 min (*P_GI_* = 0.0238) compared with the high-GI breakfasts (Figure 2E). This resulted in higher AUC240 of desire to eat scores following both low-GI breakfasts compared to high-GI breakfasts (*P_GI_* = 0.0291) (Figure 2F).

For prospective eating, the LCLGI breakfast resulted in the highest scores compared to the other three breakfasts at 60 min (*P_GI_* = 0.0021, *P_Carbohydrate_* = 0.0334). Both low-carbohydrate breakfasts resulted in higher scores compared to the high-carbohydrate breakfasts at 120 min (*P_Carbohydrate_* = 0.0424) (Figure 2G). This resulted in the highest AUC240 of prospective eating scores in response to the LCLGI breakfast in comparison to the other three breakfasts (*P_Interaction_* = 0.0153) (Figure 2H). Detailed data on postprandial subjective satiety responses were presented in Supplementary Table S2.

Since the postprandial subjective satiety score was not the primary outcome for sample size estimation in this study, we calculated Hedges’ g values between any two of the intervention groups as a measure of effect size (Supplementary Table S3). The AUC240 of fullness scores between any two breakfasts showed negligible to medium effects, with absolute Hedges’ g values ranging from 0.01 to 0.51. The AUC240 of hunger scores between LCLGI and the other three breakfasts showed large effects for hunger AUC240, with absolute Hedges’ g values ranging from 0.84 to 0.99. The AUC240 of desire to eat and prospective eating scores between LCLGI and the other three breakfasts exhibited medium effects, with absolute Hedges’ g values ranging from 0.51 to 0.76 (Supplementary Table S3).

The concentrations of postprandial ghrelin, GLP-1, and GIP following 240 min of breakfast were similar among the four breakfasts (all *p* > 0.05) (Figure 3 and Supplementary Table S2). The calculated effect sizes of gut hormone concentrations were generally negligible to small, with absolute Hedges’ g values ranging from 0 to 0.42 (Supplementary Table S3).

### 3.3. Sensitivity Analyses of Postprandial Subjective Satiety Responses and Gut Hormone Concentrations

The leave-one-participant-out sensitivity analyses showed that the main effects of carbohydrate and GI on hunger AUC240 remained significant after the exclusion of any single participant. For other outcomes, the significance of effects varied depending on the excluded participant, which might reflect the modest sample size and the negligible to medium effect sizes of these outcomes (Supplementary Table S4).

### 3.4. Relationships Between Postprandial Subjective Satiety and Gut Hormone Concentrations

At 240 min after four breakfasts, plasma ghrelin concentrations were inversely correlated with fullness scores (r = −0.390, *p* = 0.0035) and positively correlated with hunger, desire to eat, and prospective eating scores (r = 0.287 to 0.358, all *p* < 0.05). Changes in plasma ghrelin concentrations from fasting to 240 min were inversely correlated with the AUC240 of prospective eating scores (r = −0.347, *p* = 0.0102). Both plasma GLP-1 concentrations at 240 min and their changes from fasting to 240 min were inversely correlated with the AUC240 of fullness scores (r = −0.417, *p* = 0.0017) and positively correlated with the AUC240 of hunger, desire to eat, and prospective eating scores (r = 0.343 to 0.442, all *p* < 0.05) (Figure 4A).

Changes in plasma ghrelin concentrations from fasting to 240 min were positively correlated with changes from baseline to peak of postprandial fullness scores (r = 0.287, *p* = 0.0356) and prospective eating scores (r = 0.456, *p* = 0.0005) (Figure 4B). Changes in plasma GLP-1 concentrations from fasting to 240 min were inversely correlated with changes from baseline to peak postprandial fullness scores (r = −0.296, *p* = 0.0296) (Figure 4B).

### 3.5. Subgroup Analysis Based on Sex, Habitual Carbohydrate Intake Quantity and Quality

Habitual carbohydrate intake (%E, CQI, and CF) was correlated with postprandial subjective satiety scores or gut hormone concentrations (Supplementary Figure S1). The habitual %E of carbohydrate was positively correlated with the AUC240 of fullness, whereas the CQI was negatively correlated with the AUC240 of fullness and positively correlated with the AUC240 of hunger. The CF was positively correlated with GLP-1 concentrations and their changes from fasting. No significant correlations were observed for dietary GI or GL (Supplementary Figure S1). In exploratory subgroup analyses of postprandial subjective satiety, females demonstrated higher AUC240 of hunger and desire to eat scores following low-carbohydrate vs. high-carbohydrate breakfasts (all *P_Carbohydrate_* < 0.05) (Supplementary Figure S2A).

Stratification by habitual %E of carbohydrate revealed that in comparison to high-carbohydrate breakfasts, low-carbohydrate breakfasts resulted in higher AUC240 of hunger scores in participants with lower %E (*P_Carbohydrate_* = 0.0297) and higher AUC240 of desire to eat (*P_Carbohydrate_* = 0.0495) and prospective eating scores (*P_Carbohydrate_* = 0.0183) in participants with higher %E (Supplementary Figure S2B).

In participants with lower CQI, both low-GI breakfasts resulted in higher AUC240 of hunger (*P_GI_* = 0.0214) and prospective eating scores (*P_GI_* = 0.0411) compared to both high-GI breakfasts. In participants with higher CQI, both low-carbohydrate breakfasts resulted in higher AUC240 of desire to eat scores (*P_Carbohydrate_* = 0.0398) compared to both high-carbohydrate breakfasts. Among participants with higher CQI or CF, the LCLGI breakfast consistently resulted in higher AUC240 of hunger and prospective eating scores compared with at least one of the other breakfasts (Supplementary Figure S2C,D).

For gut hormones, sex-stratified analyses revealed differential ghrelin responses. Males had lower 240 min postprandial plasma ghrelin concentrations following low-GI vs. high-GI breakfasts (*P_GI_* = 0.0318), while females had higher ghrelin concentrations following low-carbohydrate vs. high-carbohydrate breakfasts (*P_Carbohydrate_* = 0.0362) and higher plasma GLP-1 concentrations following low-GI vs. high-GI breakfasts (*P_GI_* = 0.0207) (Supplementary Figure S3A). Participants with lower CQI had lower ghrelin concentrations following low-GI vs. high-GI breakfasts (*P_GI_* = 0.0112), and participants with higher CQI had higher ghrelin concentrations following low-carbohydrate vs. high-carbohydrate breakfasts (*P_Carbohydrate_* = 0.0374) (Supplementary Figure S3C). Gut hormone concentrations were similar among the four breakfasts in subgroup analyses based on habitual %E from carbohydrate and CF (all *p* > 0.05) (Supplementary Figure S3B,D).

## 4. Discussion

To our knowledge, this acute randomized crossover controlled-feeding trial provided the first documentation of the effects of low-carbohydrate or high-carbohydrate breakfasts with low-GI or high-GI values on 240 min postprandial subjective satiety and plasma concentrations of gut hormones in healthy Chinese adults. Low-GI breakfasts resulted in lower 240 min postprandial AUC240 of fullness scores and higher AUC240 of desire to eat scores than high-GI breakfasts, and the LCLGI breakfast resulted in the highest 240 min postprandial AUC240 of hunger and prospective eating scores among the four breakfasts. These results suggested inhibitory effects of low-GI breakfasts, specifically the LCLGI breakfast, on postprandial subjective satiety, which were contradictory to our hypothesis. Across all participants, postprandial gut hormone concentrations did not differ significantly among the four breakfasts. Exploratory subgroup analyses demonstrated that sex and habitual carbohydrate intake modified the postprandial subjective satiety and gut hormone concentrations in response to breakfasts.

The leave-one-participant-out sensitivity analyses showed that the main effects of carbohydrate and GI on hunger AUC240 were robust to the exclusion of any single participant, consistent with the large size observed for these outcomes. In contrast, the effects on fullness, desire to eat, prospective eating scores, and gut hormones, which exhibited small-to-medium effect sizes, did not remain consistently significant after excluding individual participants. This differential robustness indicated limited statistical power of the current sample size (*n* = 15) for detecting small-to-medium effects, rather than spurious findings. Future studies with larger sample sizes powered for subjective satiety outcomes are warranted to confirm these findings.

In the current study, low-GI breakfasts resulted in less favorable 240 min postprandial subjective satiety responses than high-GI breakfasts, particularly for the LCLGI breakfast. These findings aligned with a previous acute controlled-feeding RCT reporting lower postprandial subjective satiety following low-GI meals than high-GI meals when the carbohydrate energy ratio was high (67%E) [46]. However, contrary to our findings, some other acute interventional studies with moderate or high-carbohydrate contents (57–64%E) or low-carbohydrate contents (40%E) have reported that low-GI meals resulted in higher fullness scores and lower hunger scores [18], or did not significantly affect fullness scores [25], hunger scores [20,25], desire to eat scores [25], or prospective eating scores [18] compared to high-GI controls. The current literature collectively has reported heterogeneous findings on the effects of low-GI versus high-GI meals with various carbohydrate contents on postprandial subjective satiety, suggesting that food properties beyond GI values may also influence satiety. In the current study, the main food sources of low-GI breakfast were soda crackers and whole wheat bread, which had high hardness. In contrast, the main food sources of high-GI breakfasts were white bread and glucose drinks, which were softer and easier to digest and absorb [47]. The differences in the physical properties of foods may directly affect the gastric emptying rates and the subsequent signal transduction of satiety [48].

Correlation analysis revealed that GLP-1 concentrations were positively correlated with hunger, desire to eat, and prospective eating scores, which was unexpected. This may be explained by the single 240 min postprandial time measurement of gut hormones, which likely reflected compensatory hormone secretion rather than causal relationships. Another possible explanation is that plasma GLP-1 concentrations may not fully reflect the local vagal or central GLP-1 signaling involved in satiety regulation [49]. In addition, the expected association between higher plasma GLP-1 concentrations and greater subjective satiety has not always been observed in human studies [50,51]. Future studies with serial measurements are needed to clarify the temporal dynamics.

Exploratory subgroup analysis revealed that sex and both the quantity and quality of habitual carbohydrate intake influenced the effects of four breakfasts on postprandial subjective satiety. The habitual diet of the participants was characterized by a moderate GI of 59.9 and a daily GL of 146.1, both within the ranges reported for Chinese adults without diabetes [52]. Interestingly, the effects of GI and carbohydrate content on subjective satiety appeared to differ between males and females. Carbohydrate seemed to have a greater influence on subjective satiety in females. These effects may be related to sex differences in postprandial substrate metabolism and the effects of sex hormones on appetite regulation [53,54]. However, the sample size of each sex subgroup was small, and future studies with larger sample sizes are needed to confirm these findings. Regardless of dietary carbohydrate energy contents, both high-carbohydrate breakfasts resulted in higher postprandial subjective satiety. In our study, both high-carbohydrate breakfasts had a higher dietary fiber content than the low-carbohydrate breakfasts. A systematic review highlighted the role of dietary fiber in enhancing satiety through increased mastication, delayed gastric emptying rates, and reduced digestion and absorption [55]. This pattern remained within the low-GI breakfasts: the HCLGI breakfast contained more refined-grain starch (27.3 vs. 5.2 g/1000 kcal) as well as more fiber (20.9 vs. 10.1 g/1000 kcal) than the LCLGI breakfast. Despite the higher refined-grain content, the HCLGI breakfast resulted in higher satiety than the LCLGI breakfast. The results of participants with lower CQI and of prospective eating AUC240 in participants with higher CF were consistent with findings in all participants. In addition, the LCLGI breakfast resulted in lower subjective satiety compared to HCLGI and/or HCHGI breakfasts among participants with higher CQI. These findings collectively suggested that the LCLGI breakfast exerted inhibitory effects on postprandial hunger and prospective eating in participants, irrespective of their habitual carbohydrate intake quality. However, given the limited sample size in the subgroup analyses, these findings are exploratory and should be interpreted with caution.

We observed no significant differences in postprandial gut hormone concentrations among the four breakfasts. In all participants, the ghrelin results were consistent with previous findings [23,56]. Exploratory subgroup analysis demonstrated differential ghrelin responses based on sex and CQI. Among participants with lower CQI and among males, both low-GI breakfasts resulted in lower postprandial ghrelin concentrations compared to both high-GI breakfasts. Among participants with higher CQI and among females, both high-carbohydrate breakfasts resulted in lower ghrelin concentrations compared to both low-carbohydrate breakfasts. In females, both low-GI breakfasts also resulted in higher GLP-1 concentrations than both high-GI breakfasts. By design, the low-GI and high-carbohydrate breakfasts had higher dietary fiber content than high-GI and low-carbohydrate breakfasts, respectively. Previous studies have reported that dietary fiber inhibits ghrelin secretion via mechanisms such as delayed gastric emptying and short-chain fatty acid production [57,58]. These findings collectively suggested that the quality of habitual carbohydrate intakes might influence the individuals’ acute ghrelin responses to both quantity and quality of meal carbohydrate. Of note, previous studies have reported that GLP-1 and GIP are secreted rapidly, within 5–15 min after food intake, and return to baseline levels within 240 min [59,60,61]. Therefore, postprandial concentrations measured at 240 min may not capture these rapid fluctuations.

A major strength of this study was the establishment of a rigorous randomized crossover controlled-feeding trial to investigate the effects of low-carbohydrate or high-carbohydrate breakfasts with low-GI or high-GI values on 240 min postprandial subjective satiety and plasma concentrations of gut hormones. The additional effect sizes and leave-one-participant-out sensitivity analyses corroborated each other and provided further support for the observed results. The exploratory subgroup analyses were conducted based on sex and quantity and quality of habitual dietary carbohydrate intake to elucidate their modifiable effects on the observed differential responses to different breakfasts. However, some limitations must be acknowledged. By design, the breakfasts differed not only in estimated GI but also in other compositional characteristics, including dietary fiber content and fat composition. Because meal composition may influence postprandial glycemic responses and meal GI/GL determinations, and meal GI was estimated rather than directly measured, the observed effects cannot be attributed exclusively to GI [62]. In addition, VAS was measured every hour following breakfast consumption, which may not capture more subtle satiety responses, especially in the first hour after eating. Glycated hemoglobin and gastric emptying were not measured. The lack of differences in postprandial concentrations of gut hormones might be due to limited statistical power for these parameters, as this was a secondary analysis of an RCT with the sample size calculation based on the primary outcome of postprandial glycemic responses. The modest sample size limited statistical power and the precision of effect size estimates. The correlation and subgroup analyses were exploratory and involved small subgroup sample sizes. As an acute RCT, this study was not able to capture the dynamic and long-term effects of dietary interventions on postprandial subjective satiety and gut hormone concentrations. Gut hormone concentrations were measured only at a single 240 min postprandial time point, which may not adequately capture their dynamic secretion.

## 5. Conclusions

In conclusion, this study demonstrated that low-GI breakfasts resulted in lower AUC240 of fullness scores and higher AUC240 of desire to eat scores than high-GI breakfasts. The LCLGI breakfast resulted in the highest AUC240 of hunger and prospective eating scores among the four breakfasts. No significant differences in postprandial ghrelin, GLP-1, and GIP concentrations were observed among the four breakfasts. In contrast to our hypothesis and previous reports, these findings suggested that a relatively unfavorable effect of the LCLGI breakfast on postprandial satiety, without influencing postprandial plasma ghrelin, GLP-1, or GIP concentrations.

## Figures and Tables

**Figure 1:**
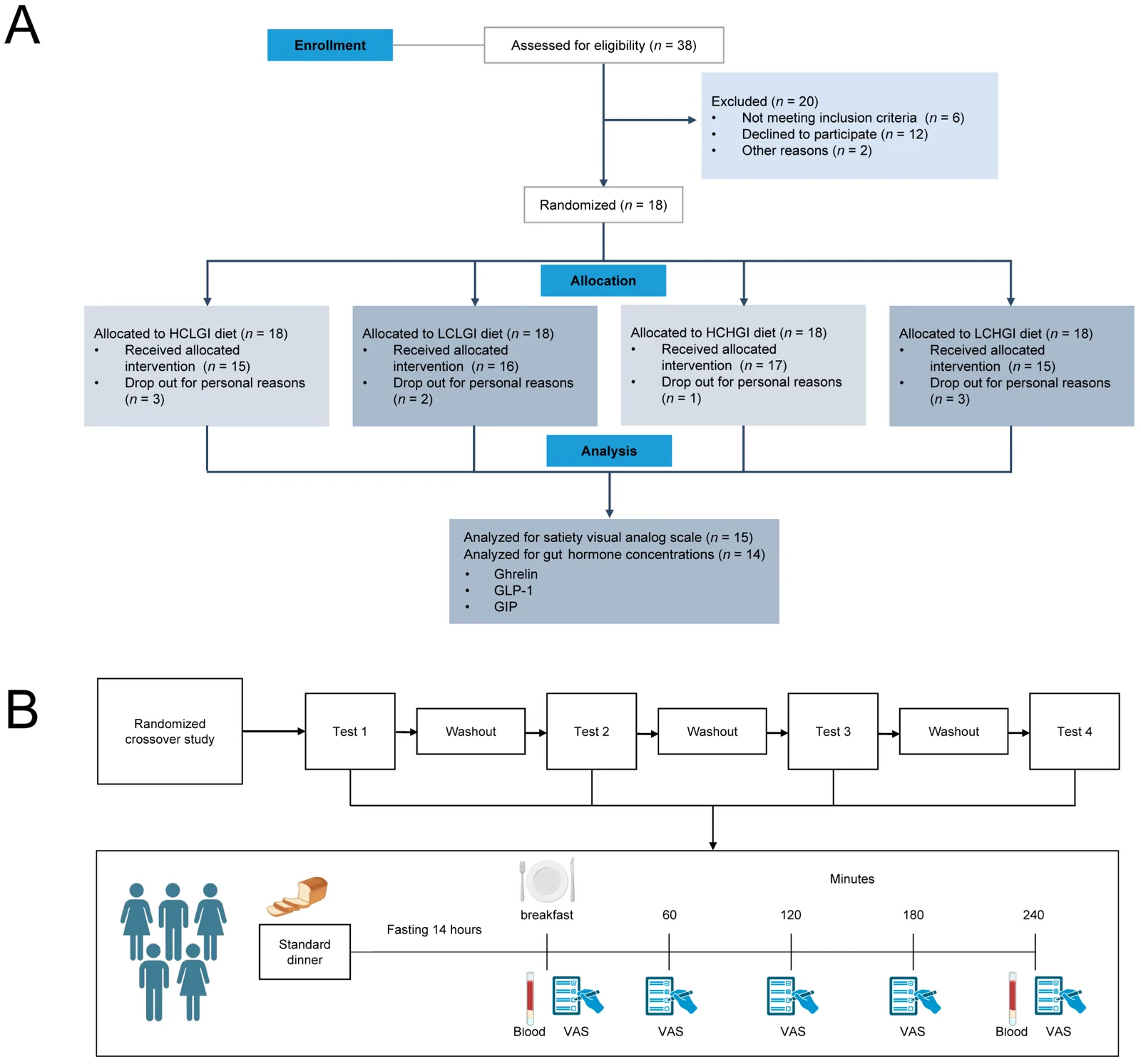
Participant flow diagram (**A**) and study design (**B**) of the acute randomized crossover controlled-feeding trial. GI, glycemic index; GIP, glucose-dependent insulinotropic polypeptide; GLP-1, glucagon-like peptide-1; HCHGI, high-carbohydrate meal with high-GI value; HCLGI, high-carbohydrate meal with low-GI value; LCHGI, low-carbohydrate meal with high-GI value; LCLGI, low-carbohydrate meal with low-GI value; VAS, visual analog scale.

**Figure 2:**
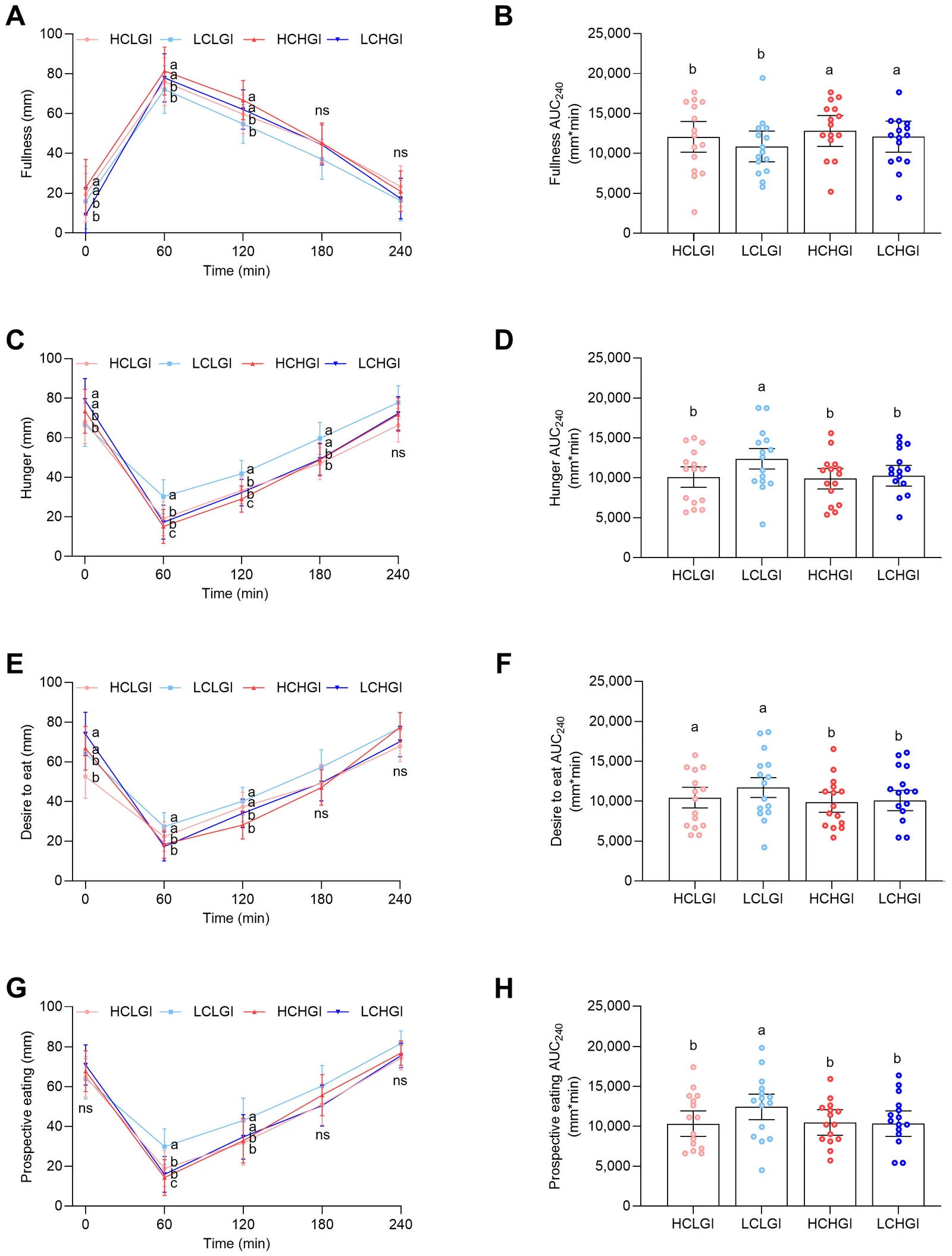
Postprandial subjective satiety responses of fullness (**A**), AUC240 of fullness (**B**), hunger (**C**), AUC240 of hunger (**D**), desire to eat (**E**), AUC240 of desire to eat (**F**), prospective eating (**G**), and AUC240 of prospective eating (**H**) during 240 min following the low-carbohydrate and high-carbohydrate breakfasts with low-GI and high-GI values. Data were presented as least-squares means ± 95% confidence limits. Different letters (a, b, or c) indicated significant differences from each other. *n* = 15. AUC, area under the curve; GI, glycemic index; HCHGI, high-carbohydrate meal with high-GI value; HCLGI, high-carbohydrate meal with low-GI value; LCHGI, low-carbohydrate meal with high-GI value; LCLGI, low-carbohydrate meal with low-GI value; ns, not significant (*p* ≥ 0.05).

**Figure 3:**
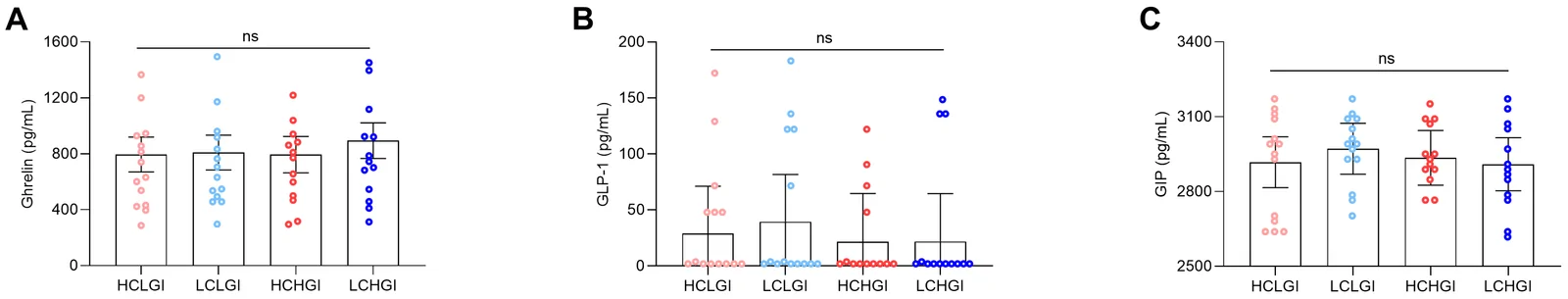
Postprandial concentrations of gut hormones, including ghrelin (**A**), GLP-1 (**B**), and GIP (**C**) at 240 min following the low-carbohydrate and high-carbohydrate breakfasts with low-GI and high-GI values. Data were presented as least-squares means ± 95% confidence limits. *n* = 14. GI, glycemic index; GIP, glucose-dependent insulinotropic polypeptide; GLP-1, glucagon-like peptide-1; HCHGI, high-carbohydrate meal with high-GI value; HCLGI, high-carbohydrate meal with low-GI value; LCHGI, low-carbohydrate meal with high-GI value; LCLGI, low-carbohydrate meal with low-GI value; ns, not significant (*p* ≥ 0.05).

**Figure 4:**
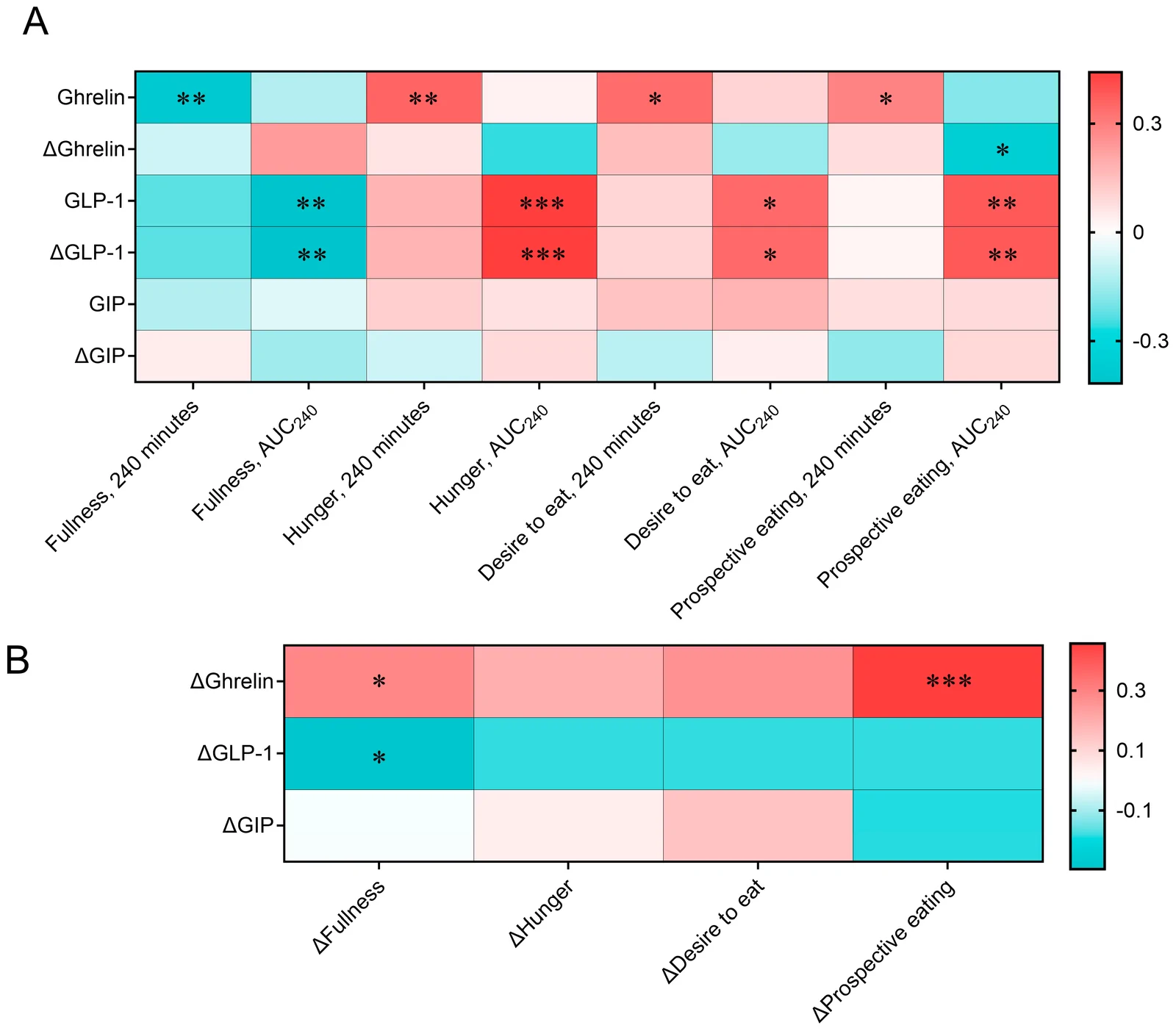
Correlations between postprandial subjective satiety responses and gut hormone concentrations at 240 min and their changes from fasting (**A**), and correlations between changes from baseline to peak postprandial VAS scores and changes in gut hormone concentrations from fasting to 240 min (**B**) following the low-carbohydrate and high-carbohydrate breakfasts with low-GI and high-GI values. Data were presented as Pearson or Spearman correlation coefficients based on the distribution of the variables. *n* = 15. *, *p* < 0.05; **, *p* < 0.01; ***, *p* < 0.001. AUC, area under the curve; GI, glycemic index; GIP, glucose-dependent insulinotropic polypeptide; GLP-1, glucagon-like peptide-1; VAS, visual analog scale.

**Table 1:** Nutrient composition of four breakfasts in the acute randomized crossover controlled-feeding trial.

Diet Variables	HCLGI	LCLGI	HCHGI	LCHGI
%Energy				
Carbohydrate	65	45	65	45
Protein	20	20	20	20
Fat	15	35	15	35
SFA	1.2	9.7	1.5	7.7
MUFA	5.9	18.8	4.2	18.3
PUFA	2.7	2.4	2.4	3.2
Dietary GI	45	45	77	77
Dietary GL	39.0	27.4	70.2	48.0
Per 1000 kcal (g)				
Fiber	20.9	10.1	6.2	3.3
Starch, whole grain	89.3	62.6	0	0
Starch, refined grain	27.3	5.2	92.7	64.8
Total sugar	4.3	5.1	29.7	19.6

GI and GL values were calculated using glucose as a reference food. GI, glycemic index; GL, glycemic load; HCHGI, high-carbohydrate meal with high-GI value; HCLGI, high-carbohydrate meal with low-GI value; LCHGI, low-carbohydrate meal with high-GI value; LCLGI, low-carbohydrate meal with low-GI value; MUFA, monounsaturated fatty acid; PUFA, polyunsaturated fatty acid; SFA, saturated fatty acid.

**Table 2:** Baseline characteristics of study participants in the acute randomized crossover controlled-feeding trial.

Variables	Total (*n* = 15)	Male (*n* = 6)	Female (*n* = 9)
Age, y	22 ± 2	23 ± 2	21 ± 2
Height, cm	163.8 ± 6.3	169.3 ± 5.1	160.1 ± 4.0
Weight, kg	64.5 ± 6.2	69.8 ± 3.6	61.0 ± 4.9
BMI, kg/m^2^	24.0 ± 1.7	24.4 ± 1.6	23.8 ± 1.8
Waist circumference, cm	80.2 ± 4.2	80.1 ± 3.9	80.3 ± 4.7
Hip circumference, cm	97.8 ± 3.3	97.4 ± 3.3	98.0 ± 3.5
Waist-hip ratio	0.82 ± 0.04	0.82 ± 0.05	0.82 ± 0.04
Blood pressures, mmHg			
Systolic blood pressure	105.1 ± 6.9	110.0 ± 4.2	101.9 ± 6.5
Diastolic blood pressure	67.6 ± 5.5	70.5 ± 5.3	65.6 ± 4.9
Physical activity			
High	2 (13.3%)	1 (16.7%)	1 (11.1%)
Moderate	7 (46.7%)	4 (66.6%)	3 (33.3%)
Low	6 (40.0%)	1 (16.7%)	5 (55.6%)
Habitual dietary intake			
Total energy, kcal	2113.2 ± 139.6	2131.8 ± 168.0	2100.9 ± 126.6
Carbohydrate, %E	50.3 ± 10.0	45.1 ± 12.0	53.8 ± 7.2
Protein, %E	16.0 ± 2.9	17.5 ± 4.0	15.0 ± 1.4
Fat, %E	33.5 ± 7.6	37.1 ± 8.7	31.1 ± 6.1
Dietary fiber, g	11.7 ± 3.8	10.7 ± 4.4	12.3 ± 3.5
Indicator of carbohydrate quality			
Dietary GI	59.9 ± 6.4	63.3 ± 6.9	57.7 ± 5.2
Dietary GL	146.1 ± 35.6	138.5 ± 40.6	151.3 ± 33.4
CQI	11.3 ± 2.5	10.2 ± 1.5	12.1 ± 2.8
CF	25.8 ± 10.2	25.9 ± 10.7	25.8 ± 10.5
Fasting glucose concentrations, mmol/L	5.2 ± 0.3	5.2 ± 0.4	5.2 ± 0.3
Fasting gut hormone concentrations, pg/mL			
Ghrelin	570.6 ± 218.1	465.3 ± 114.1	649.6 ± 249.8
GLP-1	N/A	N/A	N/A
GIP	2948.1 ± 163.9	2960.3 ± 189.7	2939.0 ± 154.8

Data were presented as mean ± SD or *n* (%). Physical activity levels were assessed using IPAQ. Data on nutrients, dietary GI, dietary GL, and CF ratio were presented after energy-adjusted intake. N/A, not detected in any sample. BMI, body mass index; CF, carbohydrate to fiber ratio; CQI, carbohydrate quality index; E, energy; GI, glycemic index; GIP, glucose-dependent insulinotropic polypeptide; GL, glycemic load; GLP-1, glucagon-like peptide-1; IPAQ, International Physical Activity Questionnaire; SD, standard deviation.

## Data Availability

Data will be made available on request.

## Supplementary Materials

The following supporting information can be downloaded at https://www.mdpi.com/article/10.3390/nu18213632/s1, Table S1: Food composition and content of four breakfasts in the acute randomized crossover controlled-feeding trial; Table S2: Main and interaction effects of carbohydrate and GI on postprandial subjective satiety responses and gut hormone concentrations (*n* = 15); Table S3: The calculated Hedges’ g for AUC240 of postprandial subjective satiety responses and gut hormone concentrations following the low-carbohydrate and high-carbohydrate breakfasts with low-GI and high-GI values (*n* = 15); Table S4: Leave-one-participant-out sensitivity analyses for the effects of AUC240 of postprandial subjective satiety and gut hormone concentrations following the low-carbohydrate and high-carbohydrate breakfasts with low-GI and high-GI values (*n* = 15); Figure S1: Correlations of habitual dietary carbohydrate intake with postprandial subjective satiety responses and gut hormone concentrations during 240 min following the low-carbohydrate and high-carbohydrate breakfasts with low-GI and high-GI values; Figure S2: Subgroup analysis of postprandial subjective satiety AUC240 following the low-carbohydrate and high-carbohydrate breakfasts with low-GI and high-GI values, stratified by sex (A), habitual percentage of energy from carbohydrate (B), CQI (C), and CF (D); Figure S3: Subgroup analysis of postprandial gut hormone concentrations at 240 min following the low-carbohydrate and high-carbohydrate breakfasts with low-GI and high-GI values, stratified by sex (A), habitual percentage of energy from carbohydrate (B), CQI (C), and CF (D).

## Abbreviations

The following abbreviations were used in this manuscript:

GLP-1	glucagon-like peptide-1
GIP	glucose-dependent insulinotropic polypeptide
RCT	randomized controlled trial
%E	percent of energy
GI	glycemic index
AUC	area under the curve
BMI	body mass index
HCLGI	high-carbohydrate meal with low-GI value
LCLGI	low-carbohydrate meal with low-GI value
HCHGI	high-carbohydrate meal with high-GI value
LCHGI	low-carbohydrate meal with high-GI value
VAS	visual analog scale
IPAQ	International Physical Activity Questionnaire
CV	coefficient of variation
FFQ	food frequency questionnaire
GL	glycemic load
CF	carbohydrate to fiber ratio
CQI	carbohydrate quality index
LSmeans	least-squares means
CL	confidence limit
SD	standard deviation
