# Supplementary material for: Effects of Breakfasts with Different Carbohydrate Contents and Glycemic Index Values on Postprandial Subjective Satiety and Plasma Gut Hormone Concentrations in Healthy Adults: An Exploratory Secondary Analysis of an Acute Randomized Crossover Controlled-Feeding Study
Source: Nutrients. 2026 Oct 31;18(21):3632. doi: 10.3390/nu18213632 (PMC13764956; doi:10.3390/nu18213632)
Supplement: Supplementary file 1 [file nutrients-18-03632-s001.zip › nutrients-4605706-supplementary.pdf]

Supplementary Table S1. Food composition and content of four breakfasts in the acute randomized crossover controlled-feeding trial.

Breakfast	Composition	Food content (g)	
		Male	Female
HCLGI	Whole wheat bread	63	50
	Soda cracker	80	63
	Chicken breast	25	20
	Cherry tomato	116	92
	Skim milk	155	123
	Apple	60	47
LCLGI	Whole wheat bread	75	59
	Soda cracker	15	12
	Chicken breast	48	38
	Cherry tomato	70	55
	Olive oil	13	10
	Milk	227	179
	Apple	65	51
HCHGI	White bread	138	109
	Chicken breast	52	41
	Glucose powder	15.6	12.2
	Cherry tomato	100	79
	Skim milk	148	117
	Apple	90	71
LCHGI	White bread	96	76
	Chicken breast	68	54
	Olive oil	12	9.5
	Glucose powder	10	7.8
	Cherry tomato	40	32
	Milk	144	114
	Apple	55	43

Specific product sources included: whole wheat bread (Keeplean® low-temperature whole wheat bread), soda cracker (DGI® soda cracker), and chicken breast (Keeplean® chicken breast) were from Methuselah Medical Technology (Shanghai) Co., Ltd. (Shanghai, China); milk (Yili® pure milk) and skim milk (Yili® skimmed milk) were from Inner Mongolia Yili Industrial Group Co., Ltd. (Hohhot, China); olive oil (XEAOTO® olive pomace oil) was from Hebei Qianhou International Trade Co., Ltd. (Handan, China); white bread (DALIYUAN® Tangzhong bread) was from Dali Foods Group Co., Ltd. (Quanzhou, China); glucose powder (Renhe® pure glucose) was from Renhe Pharmacy Co., Ltd. (Jiujiang, China); and cherry tomato and apple were obtained from local marketplace. GI, glycemic index; HCHGI, high-carbohydrate meal with high-GI value; HCLGI, high-carbohydrate meal with low-GI value; LCHGI, low-carbohydrate meal with high-GI value; LCLGI, low-carbohydrate meal with low-GI value.

Supplementary Table S2. Main and interaction effects of carbohydrate and GI on postprandial subjective satiety responses and gut hormone concentrations (*n* = 15).

Outcome	HCLGI	LCLGI	HCHGI	LCHGI	$P_{Carbohydrate}$	P_{GI}	$P_{Interaction}$
Fullness, fasting	19.39 [a]	15.95 [b]	22.87 [a]	9.14 [b]	0.0490	0.68	0.23
Fullness, 60 min	75.97 [b]	72.07 [b]	81.40 [a]	77.91 [a]	0.18	0.0305	0.94
Fullness, 120 min	59.62 [b]	54.72 [b]	66.74 [a]	62.03 [a]	0.17	0.0299	0.98
Fullness, 180 min	44.59	37.00	45.18	44.26	0.24	0.24	0.34
Fullness, 240 min	23.43	16.26	20.95	17.32	0.06	0.78	0.51
Fullness, AUC_{240}	12060.91 [b]	10849.42 [b]	12788.16 [a]	12080.57 [a]	0.07	0.0423	0.61
Hunger, fasting	68.15 [b]	66.60 [b]	73.40 [a]	78.97 [a]	0.64	0.0410	0.41
Hunger, 60 min	19.08 [b]	30.19 [a]	15.11 [c]	17.26 [b]	0.0100	0.0016	0.07
Hunger, 120 min	33.35 [b]	41.82 [a]	29.00 [c]	32.23 [b]	0.0101	0.0029	0.23
Hunger, 180 min	46.87 [b]	59.67 [a]	48.81 [b]	49.10 [a]	0.0452	0.18	0.05
Hunger, 240 min	66.31	77.81	71.64	72.28	0.13	0.98	0.17
Hunger, AUC_{240}	10097.48 [b]	12382.22 [a]	9887.70 [b]	10260.67 [b]	0.0015	0.0051	0.0178
Desire to eat, fasting	52.61 [b]	64.19 [b]	66.84 [a]	74.09 [a]	0.05	0.0109	0.64
Desire to eat, 60 min	22.34 [a]	27.34 [a]	18.51 [b]	17.33 [b]	0.58	0.0471	0.34
Desire to eat, 120 min	37.29 [a]	40.15 [a]	28.15 [b]	34.06 [b]	0.19	0.0238	0.62
Desire to eat, 180 min	49.14	57.31	47.04	49.43	0.16	0.18	0.42
Desire to eat, 240 min	67.90	77.15	77.45	70.23	0.76	0.69	0.0133
Desire to eat, AUC_{240}	10452.43 [a]	11713.61 [a]	9861.95 [b]	10085.85 [b]	0.14	0.0291	0.27
Prospective eating, fasting	64.96	63.96	67.77	70.89	0.80	0.24	0.62

Outcome	HCLGI	LCLGI	HCHGI	LCHGI	$P_{Carbohydrate}$	P_{GI}	$P_{Interaction}$
Prospective eating, 60 min	18.91 [b]	29.84 [a]	14.36 [c]	16.00 [b]	0.0334	0.0021	0.11
Prospective eating, 120 min	31.79 [b]	43.05 [a]	32.92 [b]	34.75 [a]	0.0424	0.25	0.14
Prospective eating, 180 min	51.11	60.37	55.77	50.51	0.57	0.45	0.0441
Prospective eating, 240 min	74.51	81.71	76.89	75.65	0.28	0.49	0.13
Prospective eating, AUC_{240}	10324.82 [b]	12427.32 [a]	10474.09 [b]	10333.29 [b]	0.0335	0.0316	0.0153
Ghrelin (*n* = 14)	794.75	808.04	793.07	893.30	0.15	0.27	0.26
GLP-1 (*n* = 14)	29.08	39.46	21.67	21.85	0.58	0.18	0.59
GIP (*n* = 14)	2917.18	2971.27	2934.99	2909.21	0.74	0.59	0.34

Data were presented as least-squares means. Different letters (a, b, or c) indicated significant differences from each other. AUC, area under the curve; GI, glycemic index; GIP, glucose-dependent insulinotropic polypeptide; GLP-1, glucagon-like peptide-1; HCHGI, high-carbohydrate meal with high-GI value; HCLGI, high-carbohydrate meal with low-GI value; LCHGI, low-carbohydrate meal with high-GI value; LCLGI, low-carbohydrate meal with low-GI value.

Supplementary Table S3. The calculated Hedges' g for AUC_{240} of postprandial subjective satiety responses and gut hormone concentrations following the low-carbohydrate and high-carbohydrate breakfasts with low-GI and high-GI values (*n* = 15).

Outcome	Comparison	Hedges' g (95% CI)	Adjusted *P* value
Fullness, AUC_{240}	HCHGI vs. HCLGI	0.19 (-0.29, 0.67)	0.71
	HCHGI vs. LCHGI	0.19 (-0.32, 0.69)	0.76
	HCHGI vs. LCLGI	0.51 (0.03, 1.00)	0.0355
	HCLGI vs. LCHGI	-0.01 (-0.49, 0.48)	1.00
	HCLGI vs. LCLGI	0.32 (-0.17, 0.81)	0.30
	LCHGI vs. LCLGI	0.32 (-0.15, 0.80)	0.27
Hunger, AUC_{240}	HCHGI vs. HCLGI	-0.08 (-0.67, 0.50)	0.98
	HCHGI vs. LCHGI	-0.15 (-0.73, 0.43)	0.90
	HCHGI vs. LCLGI	-0.99 (-1.57, -0.41)	0.0003
	HCLGI vs. LCHGI	-0.06 (-0.65, 0.52)	0.99
	HCLGI vs. LCLGI	-0.90 (-1.48, -0.32)	0.0009
	LCHGI vs. LCLGI	-0.84 (-1.42, -0.25)	0.0024
Desire to eat, AUC_{240}	HCHGI vs. HCLGI	-0.24 (-0.98, 0.51)	0.83
	HCHGI vs. LCHGI	-0.09 (-0.81, 0.63)	0.99
	HCHGI vs. LCLGI	-0.76 (-1.47, -0.04)	0.0359
	HCLGI vs. LCHGI	0.15 (-0.63, 0.92)	0.96
	HCLGI vs. LCLGI	-0.51 (-1.25, 0.23)	0.27
	LCHGI vs. LCLGI	-0.66 (-1.37, 0.05)	0.08
Prospective eating, AUC_{240}	HCHGI vs. HCLGI	0.05 (-0.49, 0.58)	1.00
	HCHGI vs. LCHGI	0.04 (-0.49, 0.58)	1.00
	HCHGI vs. LCLGI	-0.62 (-1.15, -0.09)	0.0167
	HCLGI vs. LCHGI	0.00 (-0.53, 0.53)	1.00
	HCLGI vs. LCLGI	-0.67 (-1.21, -0.13)	0.0097
	LCHGI vs. LCLGI	-0.67 (-1.19, -0.14)	0.0080
Ghrelin (*n* = 14)	HCHGI vs. HCLGI	-0.01 (-0.63, 0.62)	1.00
	HCHGI vs. LCHGI	-0.42 (-1.06, 0.21)	0.29
	HCHGI vs. LCLGI	-0.06 (-0.68, 0.55)	0.99
	HCLGI vs. LCHGI	-0.42 (-1.03, 0.19)	0.27

Outcome	Comparison	Hedges' g (95% CI)	Adjusted *P* value
	HCLGI vs. LCLGI	-0.06 (-0.66, 0.55)	0.99
	LCHGI vs. LCLGI	0.36 (-0.23, 0.96)	0.37
GLP-1 (*n* = 14)	HCHGI vs. HCLGI	-0.09 (-0.55, 0.36)	0.95
	HCHGI vs. LCHGI	0.00 (-0.48, 0.47)	1.00
	HCHGI vs. LCLGI	-0.22 (-0.68, 0.23)	0.55
	HCLGI vs. LCHGI	0.09 (-0.36, 0.54)	0.95
	HCLGI vs. LCLGI	-0.13 (-0.57, 0.31)	0.85
	LCHGI vs. LCLGI	-0.22 (-0.66, 0.21)	0.52
GIP (*n* = 14)	HCHGI vs. HCLGI	0.09 (-0.73, 0.91)	0.99
	HCHGI vs. LCHGI	0.13 (-0.70, 0.96)	0.97
	HCHGI vs. LCLGI	-0.18 (-0.99, 0.62)	0.93
	HCLGI vs. LCHGI	0.04 (-0.77, 0.85)	1.00
	HCLGI vs. LCLGI	-0.28 (-1.08, 0.52)	0.78
	LCHGI vs. LCLGI	-0.32 (-1.11, 0.47)	0.70

Values were presented as Hedges' g with 95% confidence intervals. Adjusted *P* values were obtained using the Tukey-Kramer method. AUC, area under the curve; CI, confidence interval; GI, glycemic index; GIP, glucose-dependent insulinotropic polypeptide; GLP-1, glucagon-like peptide-1; HCHGI, high-carbohydrate meal with high-GI value; HCLGI, high-carbohydrate meal with low-GI value; LCHGI, low-carbohydrate meal with high-GI value; LCLGI, low-carbohydrate meal with low-GI value.

Supplementary Table S4. Leave-one-participant-out sensitivity analyses for the effects of AUC_{240} of postprandial subjective satiety and gut hormone concentrations following the low-carbohydrate and high-carbohydrate breakfasts with low-GI and high-GI values (*n* = 15).

Outcome	$P_{Carbohydrate}$	P_{GI}	$P_{Interaction}$
Fullness, AUC_{240}	0.0117 - 0.1945	0.0244 - 0.0888	0.3177 - 0.9294
Hunger, AUC_{240}	<0.0001 - 0.0124	0.0028 - 0.0171	0.0039 - 0.1178
Desire to eat, AUC_{240}	0.0344 - 0.3059	0.0141 - 0.0521	0.1031 - 0.7968
Prospective eating, AUC_{240}	0.0107 - 0.1078	0.0203 - 0.0887	0.0052 - 0.0719
Ghrelin (*n* = 14)	0.0444 - 0.8227	0.0300 - 0.6641	0.1595 - 0.4874
GLP-1 (*n* = 14)	0.3260 - 0.8353	0.0403 - 0.3901	0.3298 - 0.9823
GIP (*n* = 14)	0.5098 - 0.9881	0.3245 - 0.9965	0.1647 - 0.5664

Values represent the range of *P* values obtained from leave-one-participant-out sensitivity analyses. AUC, area under the curve; GI, glycemic index; GIP, glucose-dependent insulinotropic polypeptide; GLP-1, glucagon-like peptide-1.

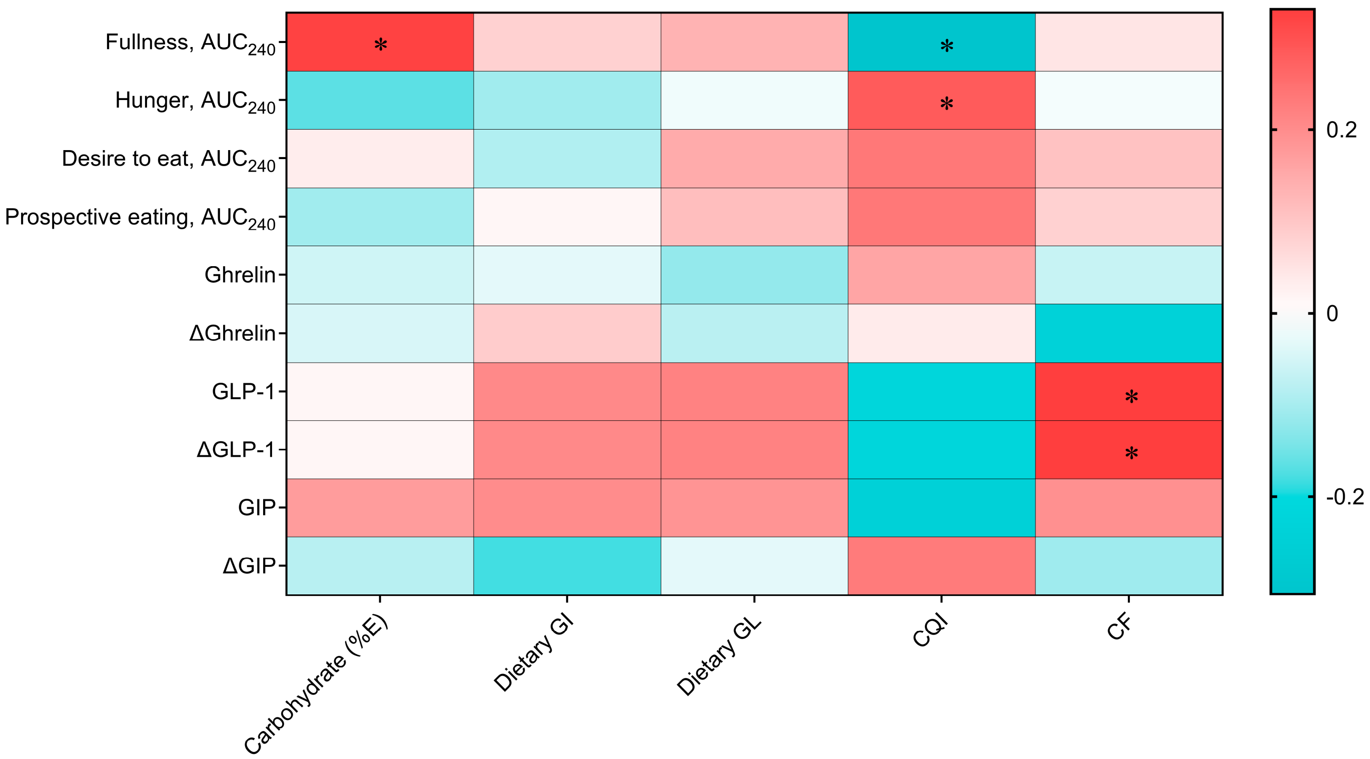

Supplementary Figure S1. Correlations of habitual dietary carbohydrate intake with postprandial subjective satiety responses and gut hormone concentrations during 240 min following the low-carbohydrate and high-carbohydrate breakfasts with low-GI and high-GI values. Data were presented as Pearson or Spearman correlation coefficients based on the distribution of the variables. n = 15. *, $P < 0.05$. AUC, area under the curve; E, energy; CF, carbohydrate to fiber ratio; CQI, carbohydrate quality index; GI, glycemic index; GIP, glucose-dependent insulinotropic polypeptide; GL, glycemic load; GLP-1, glucagon-like peptide-1.

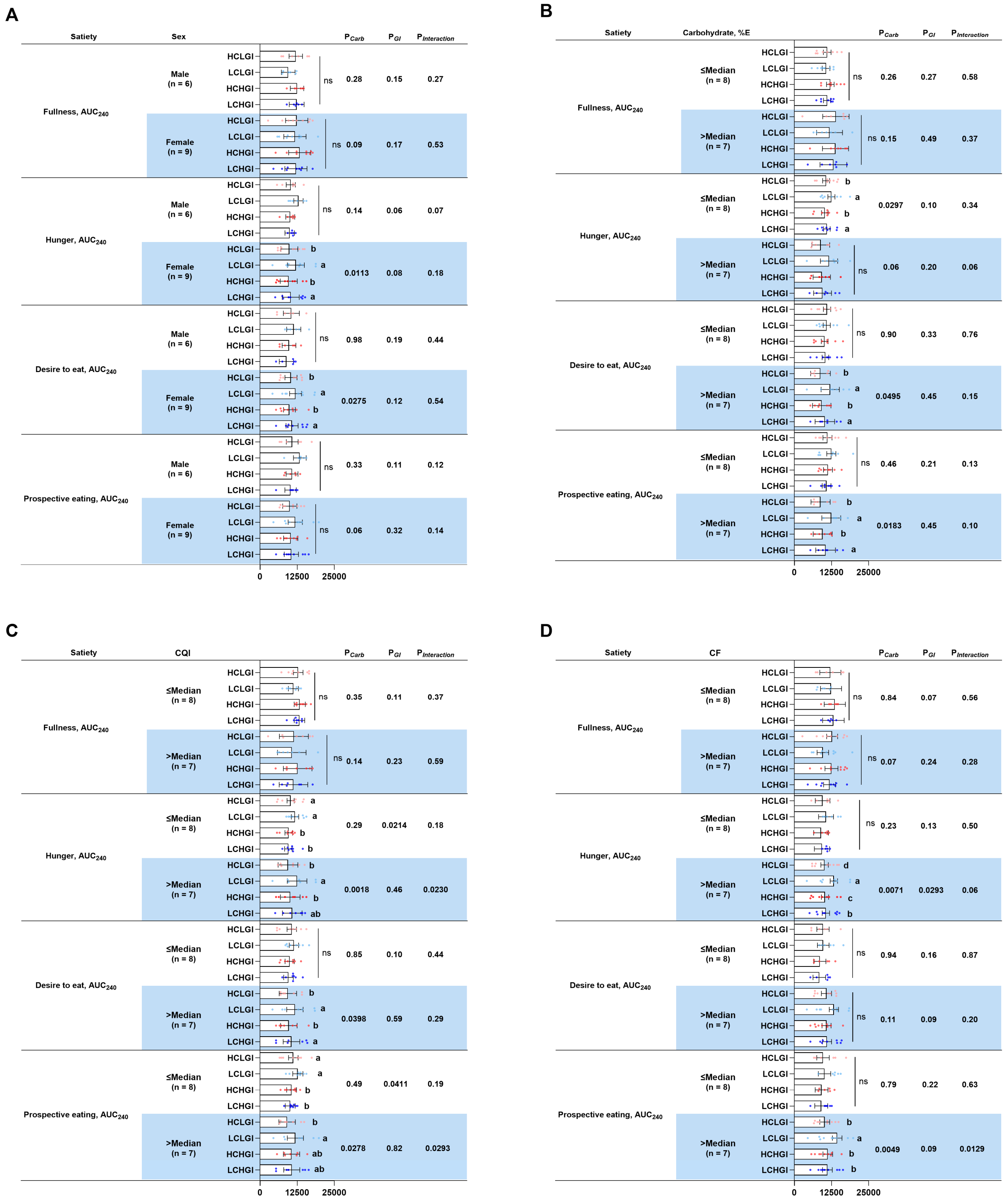

Supplementary Figure S2. Subgroup analysis of postprandial subjective satiety AUC240 following the low-carbohydrate and high-carbohydrate breakfasts with low-GI and high-GI values, stratified by sex (A), habitual percentage of energy from carbohydrate (B), CQI (C), and CF (D). Data were presented as least-squares means ± 95% confidence limits. Different letters (a or b) indicated significant differences from each other. n = 15. AUC, area under the curve; Carb, carbohydrate; CF, carbohydrate to fiber ratio; CQI, carbohydrate quality index; E, energy; GI, glycemic index; HCHGI, high-carbohydrate meal with high-GI value; HCLGI, high-carbohydrate meal with low-GI value; LCHGI, low-carbohydrate meal with high-GI value; LCLGI, low-carbohydrate meal with low-GI value; ns, not significant ($P \geq 0.05$).

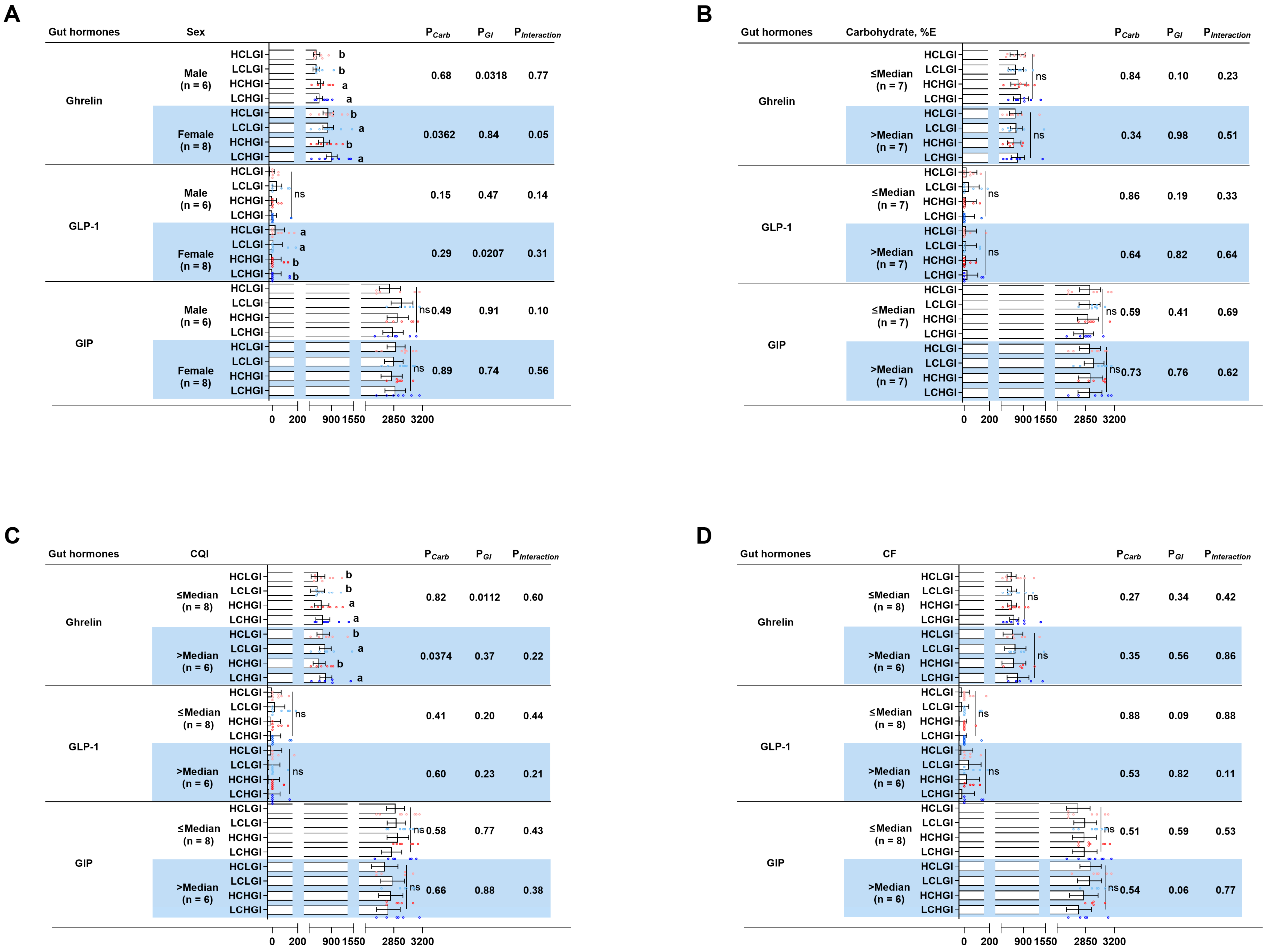

Supplementary Figure S3. Subgroup analysis of postprandial gut hormone concentrations at 240 min following the low-carbohydrate and high-carbohydrate breakfasts with low-GI and high-GI values, stratified by sex (A), habitual percentage of energy from carbohydrate (B), CQI (C), and CF (D). Data were presented as least-squares means ± 95% confidence limits. Different letters (a or b) indicated significant differences from each other. n = 14. Carb, carbohydrate; CF, carbohydrate to fiber ratio; CQI, carbohydrate quality index; E, energy; GI, glycemic index; GIP, glucose-dependent insulinotropic polypeptide; GLP-1, glucagon-like peptide-1; HCHGI, high-carbohydrate meal with high-GI value; HCLGI, high-carbohydrate meal with low-GI value; LCHGI, low-carbohydrate meal with high-GI value; LCLGI, low-carbohydrate meal with low-GI value; ns, not significant ($P \geq 0.05$).
